# In Vitro Lytic Activity and Surface Persistence of Bacteriophages and Efficacy of a Phage Cocktail Against Multidrug-Resistant Monophasic *Salmonella* Typhimurium on Different Poultry Farm Surfaces

**DOI:** 10.3390/antibiotics15100954

**Published:** 2026-09-25

**Authors:** Nicla Gentile, Laura Montoro-Dasi, Ana Marco-Fuertes, Mircea Coroian, Giuseppe D’auria, Laura Lorenzo-Rebenaque, Clara Marin

**Affiliations:** 1 Institute of Science and Animal Technology, Universitat Politècnica de València, Camí de Vera s/n, 46022 Valencia, Spain; ngentil@upv.edu.es (N.G.); 2 Facultad de Veterinaria, Instituto de Ciencias Biomédicas, Universidad Cardenal Herrera-CEU, CEU Universities, Alfara del Patriarca, 46115 Valencia, Spain; laura.montoro@uchceu.es (L.M.-D.); ana.marcofuertes@uchceu.es (A.M.-F.); 3 Department of Poultry Management and Pathology, University of Agricultural Sciences and Veterinary Medicine of Cluj-Napoca, 400372 Cluj-Napoca, Romania; mircea.coroian@usamvcluj.ro; 4 Department of Parasitology and Parasitic Diseases, University of Agricultural Sciences and Veterinary Medicine of Cluj-Napoca, 400372 Cluj-Napoca, Romania; 5 Sequencing and Bioinformatics Service, Foundation for the Promotion of Health and Biomedical Research of Valencia Region—FISABIO, 46020 Valencia, Spain; giuseppe.dauria@fisabio.es

**Keywords:** biosecurity, bacteriophage, cleaning and disinfection

## Abstract

**Background/Objectives:** Organic matter can compromise cleaning and disinfection (C&D) efficacy, making *Salmonella* control on farms challenging. This study aimed to characterize the in vitro lytic activity of individual bacteriophages (BPs) against mST and other *Salmonella* serovars of public health importance and to select BPs with complementary lytic profiles for cocktail formulation. The resulting BP cocktail was then evaluated against multidrug-resistant mST under simulated poultry farm conditions on metal, concrete, and plastic slat surfaces in the presence of organic matter, while BP persistence and viability were monitored over time. **Methods:** Individual BPs were screened for lytic activity against mST, SE, ST, SV, SH, and SI, and four BPs with complementary lytic profiles were selected and combined into a cocktail at 10^7^ PFU/mL. The resulting BP cocktail was then evaluated under simulated poultry farm conditions for its ability to control mST on metal, concrete, and plastic slats. The surfaces were then contaminated with poultry faeces artificially inoculated with mST at 10^6^ CFU/g and treated with two applications of the BP cocktail at a 24 h interval. *Salmonella* detection and BP persistence on the different surfaces were monitored throughout the experiment. **Results:** Following the first BP application, *Salmonella* detection was significantly reduced, with no *Salmonella* detected on metal surfaces. After the second application, *Salmonella* was no longer detected on any of the treated surfaces. In addition, the BP cocktail persisted on all surfaces, maintaining a titre of 5.14 log_10_ PFU/mL at the end of the experimental period. **Conclusions:** The selected BP cocktail demonstrated lytic activity against six epidemiologically important *Salmonella* serovars and reduced multidrug-resistant mST on faeces-contaminated poultry surfaces after two applications. These results support its potential use as a complementary biosanitation strategy for C&D procedures.

## 1. Introduction

*Salmonella enterica* remains one of the leading zoonotic pathogens worldwide, presenting a persistent challenge for public health and food safety [1]. Across the European Union (EU), 79,703 confirmed human salmonellosis cases were reported in 2024, a 1.7% increase from 2023 [2]. Despite this, the overall incidence of salmonellosis has decreased considerably over the last decade, mainly due to the implementation of National *Salmonella* Control Programmes (**NCPs**), which include stringent cleaning and disinfection (**C&D**) protocols in livestock production systems to control *Salmonella* from the farm to fork [2,3].

In the poultry sector, *Salmonella* Enteritidis (**SE**), *Salmonella* Typhimurium (**ST**), and the monophasic variant of *S.* Typhimurium (**mST**) remain the predominant serovars and constitute the primary targets of current control strategies. Although mST has historically been associated with the swine sector, its occurrence in poultry production has increased in recent years, leading to its inclusion among the serovars targeted by the NCPs, together with SE and ST [4]. In Europe, the number of reported human salmonellosis cases caused by mST increased from 1.738 in 2023 to 3.860 in 2024, making it the third most frequently reported serovar after SE [5]. Within the NCPs, the maximum permitted prevalence of target serovars (SE, ST, and mST) is 1% in laying hen flocks and 2% in broiler flocks [4]. In breeder flocks, however, control measures extend to additional serovars of public health importance, including *S.* Infantis (**SI**), *S.* Virchow (**SV**) and *S.* Hadar (**SH**) [2,5]. The detection of any regulated serovar (SE, ST, mST, SI, SV, or SH) requires flock culling and destruction of associated products, highlighting the epidemiological importance of controlling a broader range of *Salmonella* serovars within the poultry production pyramid [4].

Among poultry health management strategies, vaccination is an effective strategy that reduces *Salmonella* shedding and transmission in poultry [6]. However, most commercial vaccines are based on SE and ST strains, and protection against heterologous serovars may be limited [6]. This limitation becomes particularly relevant in cases of co-infection involving two or more *Salmonella* serovars. Several studies have reported the simultaneous circulation of multiple serovars within the same poultry farm [7,8,9,10]. Therefore, vaccination should be complemented by effective biosecurity measures to reduce environmental contamination and pathogen transmission between production cycles.

Most commercial poultry producers employ an “all-in, all-out” production system, ensuring thorough C&D between flocks [11]. It is generally accepted that cleaning procedures can remove approximately 90% of bacterial contamination, while subsequent disinfection allows for a further reduction in the microbial load [12,13]. However, several factors can compromise the effectiveness of these procedures, including improper disinfectant application, which can promote the development of tolerance, and the presence of organic matter [11,12,14,15]. Obe et al. (2020) reported high levels of tolerance to chlorine-based disinfectants and quaternary ammonium compounds (**QACs**) among *Salmonella* strains isolated after disinfection procedures and identified a positive correlation between disinfectant tolerance and biofilm-forming ability [12]. Additional evidence indicates that susceptibility to disinfectants may vary among different *Salmonella* serovars. Marin et al. (2009) demonstrated that SE and SV are generally more susceptible to disinfectants than ST, SH and SI, regardless of their biofilm-forming capacity [16]. According to Wales et al. (2006), the effectiveness of sanitation protocols depends more on the quality of the preliminary cleaning procedures than on the selection of the disinfectant applied, highlighting the negative impact of residual organic matter on disinfection efficacy [15].

Moreover, environmental factors such as temperature and surface type influence the effectiveness of disinfection protocols [13,16,17,18]. Poultry production systems contain a variety of surfaces, including metal, plastic, and concrete structures, as well as equipment commonly found inside poultry houses, such as drinkers, cages, and feeders [19,20]. These surfaces are characterized by distinct physicochemical properties (e.g., porosity and irregularity) that may influence the persistence and survival of enterobacteria, including *Salmonella*, making their complete elimination more difficult [13,17,21,22,23]. Several studies have shown that *Salmonella* can persist on different surface types even after C&D procedures [24,25,26].

Taken together, this evidence highlights the need to develop sanitation strategies capable of ensuring uniform efficacy against the main serovars of public health importance, particularly in the presence of organic matter on surfaces that are difficult to sanitize. Among the promising strategies is the use of bacteriophages (**BPs**), viruses that selectively infect their bacterial hosts [27]. BPs offer several advantages, including high specificity, self-replication capacity, good tolerability, and widespread availability in nature [28]. They can be applied as biocides, individually or combined into BP cocktails, to broaden their host range and enhance antibacterial efficacy [18,29]. Previous studies have evaluated BPs as a biocidal agent to control *Salmonella* in poultry production systems, proposing them as a potential alternative or complement to conventional chemical disinfectants [30,31,32]. However, limited information is available on the efficacy of BPs in the presence of organic matter on surfaces under conditions representative of livestock environments. Therefore, the present study aims to: (**i**) characterize the in vitro lytic activity of individual BPs against the mST strain and other *Salmonella* serovars of public health importance (SE, ST, SV, SH, and SI), and to select BPs with complementary lytic profiles for formulation of the BP cocktail, and (**ii**) to evaluate the efficacy of the BP cocktail against the multidrug-resistant mST strain under simulated poultry farm conditions, using representative poultry-house surface (metal, concrete, and plastic slats) in the presence of organic matter, and to assess the persistence and viability of the BPs on these surfaces over time.

## 2. Results

### 2.1. In Vitro Optimization of a BP Cocktail Against Public Health Salmonella Serovars

#### 2.1.1. Antimicrobial Profile of *Salmonella* Strains

The *Salmonella* strains selected for their public health importance were evaluated for antimicrobial resistance (**AMR**) and multidrug-resistant (**MDR**) profiles. All strains exhibited resistance to at least one of the 15 antibiotics tested. Overall, 3 of 6 strains (mST, SV and SI) were classified as MDR (Table 1).

The mST strain was resistant to 3 of the 15 antibiotics tested, with an AMR profile comprising SME, AMP, and TET, each stemming from three distinct antimicrobial classes: folate pathway inhibitors, **FOL**; penicillins, **PEN**; and tetracyclines, **TET** (Table 1). The mST remained susceptible to the remaining antibiotics tested: AMI, GEN, CHL, MER, CTA, CTZ, TRI, TIG, AZI, COL, CIP, and NAL. The SV strain was resistant to 4 of the 15 antibiotics tested, with an AMR profile comprising CTZ, TIG, CIP, and NAL, representing three antimicrobial classes: cephalosporins (**CEF**), quinolones (**QUI**) and glycylcyclines (**GLI**) (Table 1). Finally, the SI strain was resistant to 5 of the 15 antibiotics, showing resistance to SME, TRI, CIP, NAL and TET, with an MDR profile characterized by QUI, FOL and TET (Table 1). The SI strain remained susceptible to the remaining antibiotics tested.

#### 2.1.2. Characterization of Bacteriophages

All individual BPs from the CEU-UCH phage library were tested for their lytic activity against the six *Salmonella* strains. Lytic activity was assessed based on the intensity of bacterial lysis observed in the spot assay (Figure 1). Overall, the BPs exhibited heterogeneous lytic profiles. Φ CEU1 showed a semitransparent lysis zone against SV and a blurred zone against ST, whereas Φ CEU5 and Φ CEU12 produced a semitransparent lysis zone against SV and a blurred lysis zone against ST and mST. Φ CEU3 and Φ CEU7 displayed blurred lysis zones against ST, mST, and SI, whereas clear lysis zones were observed against SV. In contrast, Φ CEU1αP2_0402 showed a broader lytic profile, producing clear lysis zones against SE and ST, while no detectable plaque formation was observed against SV and semitransparent or blurred lysis zones were observed against the remaining *Salmonella* strains. Finally, Φ CEU9αP2_2802 produced clear lysis zones against SE and ST and a semitransparent lysis zone against mST, whereas no detectable plaque formation was observed against SI, SH, and SV.

Based on the results obtained, Φ CEU3 and Φ CEU7 were selected as additional BPs and combined with the two previously selected BPs, Φ CEU1P2_0402 and Φ CEU9P2_2802. These four BPs, highlighted in bold in Figure 2, were used to formulate the BP cocktail for the subsequent experiment.

Regarding the characterization of the two selected BPs, Φ CEU3 and Φ CEU7 showed good stability under the different stress conditions tested. In the pH stability assay, Φ CEU3 maintained a mean stability of 97.8% over the pH range from 3 to 11. However, complete inactivation was observed at pH 2, while exposure to pH 12 resulted in a 32.1% reduction in phage stability (Figure 2A). Similarly, Φ CEU7 maintained a mean stability of 94.3% between pH 3 and 9, whereas complete inactivation occurred at pH 2. Under more alkaline conditions (pH 10–12), Φ CEU7 showed a mean reduction in stability of 43.0% (Figure 2D). Thermal stability assays showed that Φ CEU3 remained fully stable after 1 h of exposure at 4 °C, whereas complete inactivation was observed at temperatures of 80 °C and above (Figure 2B). In contrast, Φ CEU7 titers decreased markedly after exposure to temperatures ranging from 60 °C to 80 °C (91.0% and 24.3%, respectively), with complete inactivation at 100 °C (Figure 2E). Finally, one-step growth curve analyses revealed eclipse periods of approximately 30 min for Φ CEU3 and 20 min for Φ CEU7 (Figure 2C and Figure 2F, respectively).

Regarding BP genome sequencing, whole-genome sequencing using the Illumina platform revealed that BP Φ CEU3 comprised a single genome of 45,056 bp, with a GC content of 46.0% and a high coding density of 95.8%. CheckV classified the genome as high-quality, with an estimated completeness of 100% based on the presence of direct terminal repeats (**DTRs**), and detected no contamination, host-derived regions, or proviral regions. The results from the VIBRANT program supported a lytic lifestyle. Pharokka annotation identified 100 coding sequences (**CDSs**) and one tRNA, identified as tRNA-Ser(UCG). The annotated CDSs included genes associated with head and genome packaging (*n* = 5), tail structures (*n* = 6), lysis (*n* = 4), DNA/RNA and nucleotide metabolism (*n* = 8), auxiliary metabolic functions and host takeover (*n* = 3), and other functions (*n* = 1), whereas 72 CDSs were assigned an unknown function (Figure 3A).

BP Φ CEU7 comprised a genome of 86,171 bp, with a GC content of 39.0% and a coding density of 90.9%. CheckV classified the genome as high-quality, with an estimated completeness of 93.9%, and detected no contamination, host-derived regions, or proviral regions. The results from the VIBRANT program also supported a lytic lifestyle. Pharokka identified 146 CDSs and 21 predicted tRNA features, of which 17 were annotated as intact tRNAs and 4 as tRNA pseudogenes. The CDSs included genes involved in head and genome packaging (*n* = 6), tail structures (*n* = 12), lysis (*n* = 4), DNA/RNA and nucleotide metabolism (*n* = 14), auxiliary metabolic functions and host takeover (*n* = 3), transcriptional regulation (*n* = 1), and other functions (*n* = 9), whereas 96 CDSs were assigned an unknown function (Figure 3B).

Moreover, no antimicrobial resistance genes or virulence-associated genes were detected in either BPs (Φ CEU3 and Φ CEU7) using the CARD and VFDB databases, respectively. In addition, Pharokka identified no genes associated with integration or excision.

Comparative genomic analysis placed both BPs within the class *Caudoviricetes*. The result from VIRIDIC analysis showed that Φ CEU3 shared the highest intergenomic similarities with *Salmonella* phages INT55 (93.6%) and INT59 (93.6%) (Figure 4A). Φ CEU3, INT55, and INT59 were assigned to the same genus-level cluster, consistent with the genus *Skatevirus*. For Φ CEU7, high intergenomic similarities were observed with *Shigella* phage JK55 (89.2%) and *Escherichia* phage allfine (89.1%) (Figure 4B). All genomes included in the Φ CEU7, VIRIDIC comparison were assigned to the same genus-level cluster, consistent with the genus *Felixounavirus*.

The complete genome sequences of Φ CEU3 and Φ CEU7 have been deposited in GenBank under accession numbers **SUB16421258** and **SUB16421315**, respectively.

### 2.2. Evaluation of Bacteriophage Cocktail Under Simulated Poultry Farm Conditions

After mST faecal artificial contamination of the surfaces (T0), *Salmonella* was present on all sampled surfaces (metal, concrete and plastic slat). Following the first application of the BP cocktail (T1), a reduction in *Salmonella* detection was observed on concrete and plastic slat surfaces in the BP-treated group (0.33 ± 0.2; 1/3) (*p* < 0.05), while mST was not detected on metal surfaces (0/3; *p* < 0.05). After the second application of the BP cocktail and 5 days after the second application of the BP cocktail (T2 and T3, respectively), no *Salmonella* was detected on any of the different surfaces in the BP-treated group (0/3; *p* < 0.05) (Figure 5).

#### Bacteriophage Stability on Surfaces

Regarding the stability of the BP cocktail on contaminated surfaces over time, BP titers decreased gradually throughout the experimental period (5.14 log_10_ PFU/mL), indicating good BP stability across all three surfaces (Figure 6).

## 3. Discussion

The *Salmonella* control in poultry farms remains a significant challenge despite the continuous implementation and updating of C&D protocols to meet production and health requirements [33]. Multiple factors influence the persistence of *Salmonella* in poultry environments, including the presence of organic matter, the physicochemical characteristics of surfaces, and the increasing spread of strains resistant to antimicrobials and disinfectants [18,34]. Consequently, there is a growing need to develop alternative or complementary sanitation strategies capable of improving pathogen control under field conditions.

In the present study, an optimized BP cocktail targeting the selected *Salmonella* serotypes was developed. The efficacy of the cocktail against a multidrug-resistant mST strain was subsequently evaluated under simulated poultry farm conditions using poultry surfaces artificially contaminated with faeces, and its stability over time.

Regarding antimicrobial susceptibility, three of the six *Salmonella* strains included in the study were classified as MDR, including the mST strain used in the simulated poultry farm contamination model. The resistance profiles identified included antimicrobials commonly used in veterinary and human medicine, such as tetracyclines, sulfonamides, fluoroquinolones, and tigecycline. The emergence of MDR *Salmonella* represents a growing public health concern because it limits available therapeutic options and increases the risk of pathogen dissemination throughout the food chain [5]. The presence of MDR strains in poultry environments further reinforces the need to develop control strategies that are not primarily dependent on antibiotic use [35]. In this context, lytic BPs represent a promising antimicrobial alternative. Furthermore, the possible co-occurrence of multiple *Salmonella* serovars within a poultry farm highlights the need for control strategies with activity against epidemiologically relevant serovars. Therefore, four BPs with complementary lytic profiles were selected in the present study to increase the spectrum of activity of the BP treatment. The spot assay results revealed differences in susceptibility among the representative strains of the six *Salmonella* serovars tested, supporting the importance of using BP cocktails rather than individual BPs when coverage of the major serotypes of public health concern is desired. BPs Φ CEU1P2_0402 and Φ CEU9P2_2802 exhibited lytic activity against SE and ST, as well as against mST and SH, whereas Φ CEU3 and Φ CEU7 expanded the spectrum of activity by effectively targeting SV and SI. Similar findings have been reported in previous studies, where BP cocktails proved more effective than individual BPs in controlling heterogeneous *Salmonella* populations or different strains belonging to the same serovar [36,37,38,39,40,41].

The characterization of Φ CEU3 and Φ CEU7 further identified characteristics potentially relevant to their use in environmental applications. BPs included in this study remained stable across a broad pH range, including highly acidic and alkaline conditions. Although stability decreased at elevated temperatures, both BPs remained stable at temperatures commonly encountered in poultry production environments. In addition, the short eclipse periods observed in the one-step growth curves indicate rapid replication and efficient infection of host bacterial cells. These characteristics may be advantageous for their use as environmental biocontrol agents [32]. Furthermore, the genomic characterization of Φ CEU3 and Φ CEU7 provided additional evidence supporting their potential use as biocontrol agents. Whole-genome analysis placed both phages within the class *Caudoviricetes*, revealing genomic relationships of Φ CEU3 and Φ CEU7 with phages belonging to the genera *Skatevirus* and *Felixounavirus*, respectively. However, comparative and phylogenetic analyses revealed a distinct genomic identity of both phages compared with the most closely related reference genomes, supporting their characterization as distinct phage isolates. Both genomes also showed a high coding density and contained genes involved in the main phage functions, including viral structure assembly, genome packaging, nucleic acid metabolism, and host cell lysis, consistent with the typical genomic organization of virulent phages [42]. A relevant difference between the two phages concerned their tRNA content: Φ CEU3 encoded only one tRNA, whereas Φ CEU7 contained 17 intact tRNAs and 4 pseudogenes. Phage-encoded tRNAs promote phage replication by corresponding to codons used by the phage genome rather than those of the host [43]. In our study, the two phages originated from wastewater, and since this environment represents a rich source of host diversity, the presence of multiple tRNAs could improve phage genome replication in multiple hosts [43]. Finally, a fundamental aspect of phage use in biocontrol concerns genomic safety [44]. No antimicrobial resistance or virulence-associated genes were detected in Φ CEU3 or Φ CEU7, nor were genes involved in integration or excision processes identified. Furthermore, the absence of lysogeny-associated genes strengthens the genomic safety profile of both phages and supports their potential use in controlling *Salmonella* [42,44].

The most relevant finding of the present study was the ability of the BP cocktail to reduce mST contamination under the simulated poultry farm conditions evaluated, including the presence of organic matter. Organic matter is known to be one of the main factors limiting the efficacy of chemical disinfectants, as it can physically protect bacteria and reduce or inactivate the action of disinfectant compounds [45,46,47]. For example, Chandler-Bostock et al. (2015) reported that phenolic disinfectants retained greater efficacy in the presence of organic matter than peroxygen and glutaraldehyde-based formulations [47]. Similarly, using experimental models that simulated dry (organic material adhering to surfaces) and wet (boot dip) contamination with poultry faeces inoculated with *Salmonella*, McLaren et al. (2011) showed that disinfectant efficacy depends on application conditions [45]. Formaldehyde was effective under dry conditions but not under wet conditions, whereas several other disinfectants, including QACs, amphoteric surfactants, iodine-based formulations, peroxygen compounds, and substituted phenols, required concentrations above the manufacturers’ recommended levels to achieve complete *Salmonella* inactivation [45].

In the present study, the BP cocktail significantly reduced *Salmonella* contamination as early as after the first application, despite the presence of organic matter. These findings are relevant for areas that are difficult to reach through routine cleaning procedures, where organic matter may persist despite sanitation efforts [12,13,17].

Previous studies have also demonstrated the potential of BPs to control *Salmonella* contamination in poultry environments. Several studies using BPs against *Salmonella* in poultry environments have also reported reductions in bacterial contamination. Sevilla-Navarro et al. (2020) reported reductions of 4.55 and 3.85 log_10_ CFU/mL for SI and SE, respectively, following BP application on poultry farm surfaces [48]. Similarly, BP cocktails applied to different matrices representative of poultry farms achieved reductions of up to 2.30 and 2.31 log_10_ CFU/mL in wood shavings and plastic surfaces, respectively, highlighting the influence of the environmental matrix on BP efficacy [49]. More recently, the application of BP in commercial broiler farms with persistent SI contamination reduced *Salmonella*-positive samples from 100% after cleaning to 36% following two BP applications, while subsequent disinfection resulted in complete elimination [50]. These findings are consistent with the results of the present study and support the use of BPs as a complementary tool within conventional C&D protocols.

Another aspect of interest concerns the stability of the BP cocktail on contaminated surfaces despite a gradual decline in BP concentration over time. This environmental persistence may contribute to prolonged antibacterial activity by allowing BP to infect surviving bacterial cells or newly introduced bacteria [35]. Similarly, previous studies have highlighted that BP persistence in poultry environments represents one of the major strengths of BP biosanitation strategies [18,35,50].

Despite these promising results, the efficacy of the BP cocktail was evaluated using an artificial contamination model based on a controlled inoculum of a single multidrug-resistant mST strain. Although this experimental approach allowed the efficacy and persistence of the BP cocktail to be evaluated under standardized and reproducible conditions, it does not fully reproduce the complexity of naturally contaminated commercial poultry farms, where *Salmonella* loads may vary and different strains or serovars may coexist within heterogeneous microbial populations and environmental conditions. Therefore, further studies under commercial farm conditions are needed to validate the efficacy of the BP cocktail against naturally occurring *Salmonella* contamination and to determine its efficacy against different *Salmonella* strains and serovars under field conditions. Another aspect to consider for the long-term implementation of BP-based biosanitation strategies is the potential emergence of phage-resistant bacteria [41]. Bacteria can activate various defense mechanisms to counteract phage infection. However, unlike conventional antimicrobials, phages can adapt and evolve to overcome these bacterial defenses, providing opportunities to identify BPs capable of infecting resistant bacterial strains or to genetically modify phages to restore their infectivity [41]. Recognizing the potential emergence of phage resistance is therefore important, and several studies have proposed the use of phage cocktails as a strategy to counteract the emergence of phage-resistant bacteria [18,41,51]. Studies suggest that combining phages targeting different bacterial receptors may delay the emergence of resistance compared with the use of individual phages or cocktails composed of phages with similar receptor specificities, which may impose strong selective pressure for receptor loss and consequently promote the emergence of resistant bacterial mutants [41,52,53].

Importantly, the BP cocktail proposed in the present study represents a potential complementary biosanitation tool within C&D programs rather than a complete replacement for conventional disinfectants. Its integration into sanitation strategies could provide an additional tool for controlling residual *Salmonella* contamination, particularly in areas where organic matter persists after routine cleaning procedures.

Overall, the results of the present study demonstrate that the BP cocktail effectively reduced multidrug-resistant mST contamination on faeces-contaminated poultry surfaces, despite the presence of organic matter, with no detectable mST following the second application. The differences observed in BP efficacy and persistence across the tested surfaces highlight the importance of considering surface characteristics and environmental conditions when developing a C&D protocol. Although this study was conducted under controlled experimental conditions, these findings support the potential use of BP cocktails as a complementary biosanitation tool to conventional C&D for controlling mST in poultry production environments. Further studies are needed to evaluate the cocktail’s efficacy against the representative strains of the other *Salmonella* serovars of public health importance included in this study and to validate its efficacy under simulated poultry farm conditions.

## 4. Materials and Methods

### 4.1. In Vitro Optimization of a Bacteriophage Cocktail Against Public Health Salmonella Serovars

#### 4.1.1. Selection and Characterization of *Salmonella* Strains

The strains selected for this experiment were: mST (CEU-215S), SE (CEU-686S), ST (CEU-685S), SV (CEU-598S), SH (CEU-422S) and SI (CEU-582S). All strains were previously isolated from field-collected faecal samples according to the procedure described in ISO/TS 6579-1:2017, serotyped according to the Kauffman–White scheme [54] and stored at −80 °C at CEU-UCH until use. These strains were selected based on their persistence in the farm environment despite the implementation of routine C&D procedures, representing a relevant challenge for effective *Salmonella* control in poultry production systems.

The strains were characterized by their AMR profile. Thus, an antimicrobial susceptibility test was performed using the EU Surveillance *Salmonella*/*E. coli* EUVSEC3 Sensititre Plate (Thermo Scientific Sensititre, Madrid, Spain). The test included the following antimicrobials and their corresponding Minimum Inhibitory Concentration (MIC) susceptibility clinical breakpoints (Susceptible, S≤; Resistant, R>; expressed in mg/L): amikacin (**AMI**, 8–8 mg/L), gentamicin (**GEN**, 2–2 mg/L), chloramphenicol (**CHL**, 8–16 mg/L), meropenem (**MER**, 2–8 mg/L), cefotaxime (**CTA**, 1–2 mg/L), ceftazidime (**CTZ**, 1–4 mg/L), sulfamethoxazole (**SME**, 64–64 mg/L), trimethoprim (**TRI**, 4–4 mg/L), tigecycline (**TIG**, 0.5–0.5 mg/L), azithromycin (**AZI**, 16–16 mg/L), ampicillin (**AMP**, 8–8 mg/L), colistin (**COL**, 2–2 mg/L), ciprofloxacin (**CIP**, 0.06–0.06 mg/L), nalidixic acid (**NAL**, 8–8 mg/L), and tetracycline (**TET**, 8–8 mg/L) [32]. Sensititre plate results were analyzed according to the breakpoints established by the European Committee on Antimicrobial Susceptibility Testing (**EUCAST**) in 2025, and MDR was defined as acquired resistance to at least one agent in three or more antimicrobial classes [55].

#### 4.1.2. Selection and Characterization of Bacteriophages

During this experiment, a cocktail of four BPs was composed. This cocktail was effective against serovars of public health significance, primarily against mST (serovar selected for the subsequent artificial contamination experiment), while also being effective in vitro against SE, ST, SV, SH, and SI, for future applications under field conditions where co-infection with different serovars may occur.

For this purpose, a total of eight BPs from the CEU-UCH phage library were selected for this study (Table 2). Two BPs had been previously selected based on their efficacy against mST under field conditions [32]: Φ CEU1P2_0402 and Φ CEU9P2_2802, and were then also evaluated against the other *Salmonella* serovars. In addition, to select two additional BPs for inclusion in the cocktail, six more BPs from our library were selected and tested to evaluate their lytic activity not only against the mST strain, but also against the major *Salmonella* serovars of public health importance, including SE, ST, SV, SH, and SI.

The two BPs that showed complementary lytic activity against mST and the other target *Salmonella* strains were selected as additional BPs for inclusion in the cocktail. These two new BPs were combined with the two previous BPs (Φ CEU1P2_0402 and Φ CEU9P2_2802), resulting in the final four-phage cocktail.

The BPs were initially isolated from sewage water samples. For this purpose, 50 mL samples of sewage water were collected from urban and swine farm sources and homogenized. Samples were centrifuged at 16,000× *g* for 5 min, and the supernatant was then filtered through a 0.22 μm membrane. The filtered supernatant was screened for phages by spot assay on different *Salmonella* lawns. Briefly, 200 μL of a log-phase bacterial culture in Luria–Bertani (**LB**; Shcarlab, Madrid, Spain), adjusted to an optical density at 600 nm (OD_600_) of 0.2 (~10^8^ CFU/mL), was mixed with 5 mL of LB soft agar (0.6% agar) tempered to 50 °C and poured onto previously prepared and dried LB basal agar (1.6% agar). Plates were allowed to dry in a laminar flow hood for 5 min. Subsequently, 20 μL of the sample was spotted onto the surface of the double-layer agar and incubated at 37 ± 1 °C for 24 h. The appearance of a clear zone on the bacterial lawn, resulting from host cell lysis, indicated the presence of a specific phage. Then, lysates of single plaques were mixed in Phosphate-Buffered Saline diluent (**PBS**; Shcarlab, Madrid, Spain) and centrifuged at 5000× *g* for 5 min, and the supernatant was filtered through a 0.22 μm membrane. Each BP was purified by serial dilutions and plated on LB agar. The process was repeated three times, after which the purified BP was stored at 4 °C.

All BPs were then tested against *Salmonella* target strains by spot test assay as described above. After incubation, the plates were inspected for evidence of bacterial lysis, and the lytic activity was assessed based on the intensity of lysis observed within the spotted area using a scale ranging from 0 to 3 (0 = no plaque, 1 = blurred, 2 = semitransparent, and 3 = clear). The target strain was considered susceptible when detectable lysis was observed within the spotted area.

Finally, the two BPs selected for inclusion in the cocktail were characterized by assessing their stability under different stress conditions and their replication dynamics using a one-step growth curve, followed by genomic characterization.

Briefly, for BP characterization, thermal stability was evaluated by incubating the BP at 4, 37, 60, 80, and 100 °C, while pH stability was assessed across a pH range from 2 to 12, adjusted with HCl and NaOH (Fisher Scientific, Hampshire, UK). In both assays, BPs were subjected to different temperature and pH conditions and incubated for 1 h. After incubation, BPs concentrations were determined by serially diluting the samples (1:10) and plating them using the drop assay method in LB agar, followed by incubation at 37 ± 1 °C for 24 h. The rates of BPs pH/thermal stability were calculated using the formula: **BP stability rate (%) = BP concentration (PFU/mL) under certain conditions/initial BP concentration added (PFU/mL) × 100%**. One-step growth experiments were performed using overnight *Salmonella* cultures (10^8^ CFU/mL), and BP was added at a multiplicity of infection (**MOI**) of 0.1. The mixture was incubated at 37 °C for 5 min to allow phage adsorption. Following incubation, the sample was centrifuged at 12,000× *g* at 4 °C for 2 min, and the supernatant was discarded to innate free BPs and prevent a new infection cycle. The pellet, containing infected bacteria, was resuspended in 10 mL of LB broth and incubated at 37 °C for 1 h. Aliquots were collected at 5, 10, 15, 20, 30, 40, 50, and 60 min. Each aliquot was centrifuged at 12,000× *g* for 2 min, and the supernatant was collected and serially diluted (1:10) for plating using the drop assay method. The experiments were performed in triplicate.

For genomic characterization, the selected BPs were sequenced in the Foundation for the Promotion of Health and Biomedical Research of the Valencian Community (**FISABIO**). Phage DNA isolation and genome sequencing were performed according to Domingo-Calap et al. (2020) [56]. Briefly, BPs were treated with DNase I to remove non-encapsidated DNA, followed by extraction using a commercial kit (QIAamp Viral RNA Mini Kit, QIAGEN, Hilden, Germany). Sequencing was subsequently performed using the Illumina MiSeq (Illumina, Inc., San Diego, CA, USA). Raw sequencing reads were subjected to quality control and pre-processing prior to de novo genome assembly. Then, taxonomic Profiling and Read Pre-processing were performed. Raw reads were quality-checked using the fastp program [57], applying default filtering and tail trimming (–cut_window_size 10, –cut_mean_quality 20). Taxonomic classification and potential contamination were assessed using KMA v1.4.15 [58] against the NCBI NT database, followed by phylogenetic aggregation with CCMetagen v1.4.1 [59]. To focus on the viral fraction, host-derived reads, if any, were depleted by mapping against the *Salmonella enterica* reference genome (subsp. *enterica* serovar Typhimurium isolate VNB151-sc-2315230, Accession LT795114.1) using BWA-MEM v0.7.17-r1188 [60]. Unmapped (non-host) reads were extracted and converted to FASTQ format using SAMtools v1.20 [61] and bedtools v2.30.0 [62] for downstream de novo assembly. Filtered viral reads were downsampled using seqtk v1.4-r122 [63] program and assembled using SPAdes v4.2.0 [64] with the metaviral algorithm, employing a multi-k-mer strategy (21, 33, 55, 77, 99, 127). Assembled contigs were filtered to retain only sequences > 5000 bp. BP genomes were then submitted to a first round of annotation using Pharokka v1.8.2 [65] for functional assignment. Genome orientation was standardized using the terminase large subunit (**TerL**) gene, followed by a second round of Pharokka annotation. CDSs, tRNAs, tRNA pseudogenes, and predicted functional categories were determined from the resulting annotations. Genome quality, completeness, contamination, host-derived regions, and proviral regions were assessed using CheckV v1.0.3 [66]. Phage lifestyle was additionally evaluated using VIBRANT v1.2.1. Genome maps were generated from the Pharokka annotations to visualize the position, orientation, and functional categories of predicted CDSs and tRNA features. The genomic safety of BPs was assessed by screening the annotated genomes for antimicrobial resistance and virulence-associated genes. Antimicrobial resistance determinants were searched against the Comprehensive Antibiotic Resistance Database (**CARD**), whereas virulence-associated genes were screened against the Virulence Factor Database (**VFDB**). Screening was performed using minimum thresholds of 90% sequence identity and 60% query coverage. The Pharokka annotations were also examined for genes associated with integration and excision. For comparative genomic characterization, related reference phages were identified by comparison against the INPHARED database using the Mash-based search implemented in Pharokka. Intergenomic nucleotide similarities between two BPs and the selected reference genomes were subsequently calculated using VIRIDIC v1.1, and species- and genus-level genome clusters were determined from the resulting intergenomic similarity matrix. Whole-genome phylogenetic analyses were performed separately for both BPs, together with their respective reference phages. Complete genome sequences were aligned using MAFFT v7.490, and poorly aligned positions were removed using trimAl v1.5.rev1 with the automated1 heuristic. Maximum-likelihood phylogenetic analyses were performed using IQ-TREE v2.0.7, with the best-fitting nucleotide substitution models selected according to the Bayesian Information Criterion (**BIC**). Branch support was assessed using 1000 ultrafast bootstrap replicates and 1000 SH-like approximate likelihood ratio test (SH-aLRT) replicates. The selected models were K3Pu + F + G4 for the Φ CEU3 dataset and TN + F + G4 for the Φ CEU7 dataset. *Salmonella* phage Akira (NC054647.1) and *Citrobacter* phage Moogle (NC027293) were used as outgroups for the Φ CEU3 and Φ CEU7 phylogenetic analyses, respectively. The resulting phylogenetic trees were subsequently visualized and graphically edited using MEGA 11. Branch support values were displayed at the corresponding nodes.

The phenotypic and genomic characterization of BPs Φ CEU1P2_0402 and Φ CEU9P2_2802 was previously carried out, and the corresponding results are presented in Gentile et al. (2026) [32].

### 4.2. Evaluation of the Bacteriophage Cocktail Under Simulated Poultry Farm Conditions

#### 4.2.1. Poultry Faeces Contamination with mST

To evaluate the BP cocktail under simulated poultry farm conditions, an experiment was performed using three poultry facility surfaces (metal, concrete, and plastic slat) at the animal facilities of the Universitat Politècnica de València (UPV, Valencia, Spain).

To contaminate poultry facility surfaces, poultry faeces were artificially contaminated with mST (CEU-215S). Faeces were collected from a poultry flock confirmed to be *Salmonella*-free according to ISO/TS 6579-1:2017. Then, the mST subculture was prepared. For this purpose, the bacteria were revived by streaking on selective chromogenic agar (**ASAP**; bioMérieux^®^, Marcy-l’Étoile, France) and then incubated at 37 ± 1 °C for 24 ± 3 h to obtain isolated colonies. Subsequently, a single colony was transferred onto nutrient agar to facilitate rapid bacterial growth. The resulting culture was then used to prepare an overnight inoculum in Buffered Peptone Water (**BPW**; Scharlau^®^, Barcelona, Spain), and the bacterial concentration was standardized to an OD_600_ nm of 0.2 (~10^8^ CFU/mL), diluted to 10^7^ CFU/mL, and used to inoculate the poultry faeces, yielding a final concentration of 10^6^ CFU/mL.

#### 4.2.2. Bacteriophage Cocktail Preparation

For the preparation of the BP cocktail for the disinfection test, the two BPs against mST (Φ CEU1P2_0402 and Φ CEU9P2_2802) [32] previously selected for their activity against mST, were combined with the two BPs (Φ CEU3 and Φ CEU7), which exhibited lytic activity against the remaining *Salmonella* strains of public health importance. Briefly, the selected BPs were combined in equal volumes and titers, then diluted with water to achieve a final concentration of 10^7^ PFU/mL, as described by Gvaladze et al. (2024) [40]. The solution was freshly prepared on the day of use.

#### 4.2.3. Experimental Design

Experimental poultry-house surfaces of metal, concrete, and plastic slat were artificially contaminated with poultry faeces. Two experimental conditions were included: an untreated control group and a BP-treated group. Surfaces in both groups were contaminated with poultry faeces inoculated with mST, whereas only the BP-treated group received the BP cocktail, as described below. For each surface type and treatment condition, three independent surfaces were used as replicates.

To this end, a circular experimental area of 20 cm in diameter was delimited on each surface and divided into four equal sampling sections. The circular experimental area was contaminated with 6 g of poultry faeces artificially inoculated with mST at a concentration of 10^6^ CFU/g (Figure 7). Each sampling section corresponded to one of the four sampling times (T0, T1, T2, or T3, as described below) and was sampled only once at its predefined sampling time (Figure 7).

After contamination, the surfaces were allowed to dry completely. Two doses of the BP cocktail were then applied to the BP-treated surfaces at 24 h intervals. Samples were collected by wiping the corresponding sampling section using both sides of a sterile wipe (Whirl-Pak^®^, Scharlab, Madrid, Spain) at the following time points: (T0) 3 h after surface contamination and before the first BP cocktail application; (T1) 24 h after the first BP cocktail application and before the second application; (T2) 24 h after the second BP cocktail application; and (T3) 5 days after the second BP cocktail application.

All collected wipe samples were analyzed for *Salmonella* detection within 24 h of sampling, following ISO/TS 6579-1:2017. Briefly, samples were pre-enriched in BPW at a 1:10 (*v*/*v*) and incubated at 37 ± 1 °C for 18 ± 2 h. Pre-enriched samples were then inoculated onto Modified Semi-Solid Rappaport Vassiliadis agar plates (**MSRV**, bioMérieux^®^, Marcy l’étoile, France) and incubated at 41.5 ± 1 °C for 48 h. Plates with suspected growth were then transferred onto selective chromogenic agar (ASAP) and incubated at 37 ± 1 °C for 24 ± 3 h. Finally, to confirm the results, a biochemical test (API-20E, bioMérieux, Marcy l’Etoile, France) was performed.

Finally, the on-farm stability of the BP cocktail on different surfaces was evaluated through BP counting to monitor its stability throughout the experiment, with samples collected at the same time points as previously described (T0–T3). Briefly, 1 mL of the sample collected as reported above was diluted 1:10 in LB Miller broth, thoroughly homogenized, centrifuged (8000× *g* at 4 °C for 10 min), and filtered through 0.45 µm and 0.22 µm filters. Serial dilutions were then prepared in LB. Then, 200 μL of a log-phase culture of the bacterial suspensions in LB at an OD_600_ nm of 0.2 (~10^8^ CFU/mL) was added to 5 mL of LB agar (LB with 0.6% agar) tempered to 50 °C and poured onto previously prepared and dried LB basal agar (with 1.6% agar). After drying for 5 min under a laminar flow hood, 10 µL of each sample dilution was spotted onto the surface of the double-layer agar and incubated at 37 °C for 24 h. BP titration was performed in triplicate.

### 4.3. Statistical Analysis

For the experiment conducted under simulated poultry farm conditions, a Generalized Linear Model (**GLM**), assuming a binomial distribution for *Salmonella* detection, was used to compare the experimental groups (untreated control vs. BP-treated group) across sampling times (T0–T3). Experimental group and sampling time were included as fixed effects, with *Salmonella* detection (positive/negative) as the binary response variable. The analysis was performed separately for each surface type (metal, concrete, and plastic slat).

GLM analysis was also used for BP characterization and BP stability on surfaces following log_10_ transformation of the data. For BP stability, phage titres were analysed throughout the sampling period (T0–T3), with sampling time included as a fixed effect.

All statistical analyses were performed using SPSS software, version 27.0 (SPSS Inc., Chicago, IL, USA). Statistical significance was set at *p* < 0.05.

## Figures and Tables

**Figure 1:**
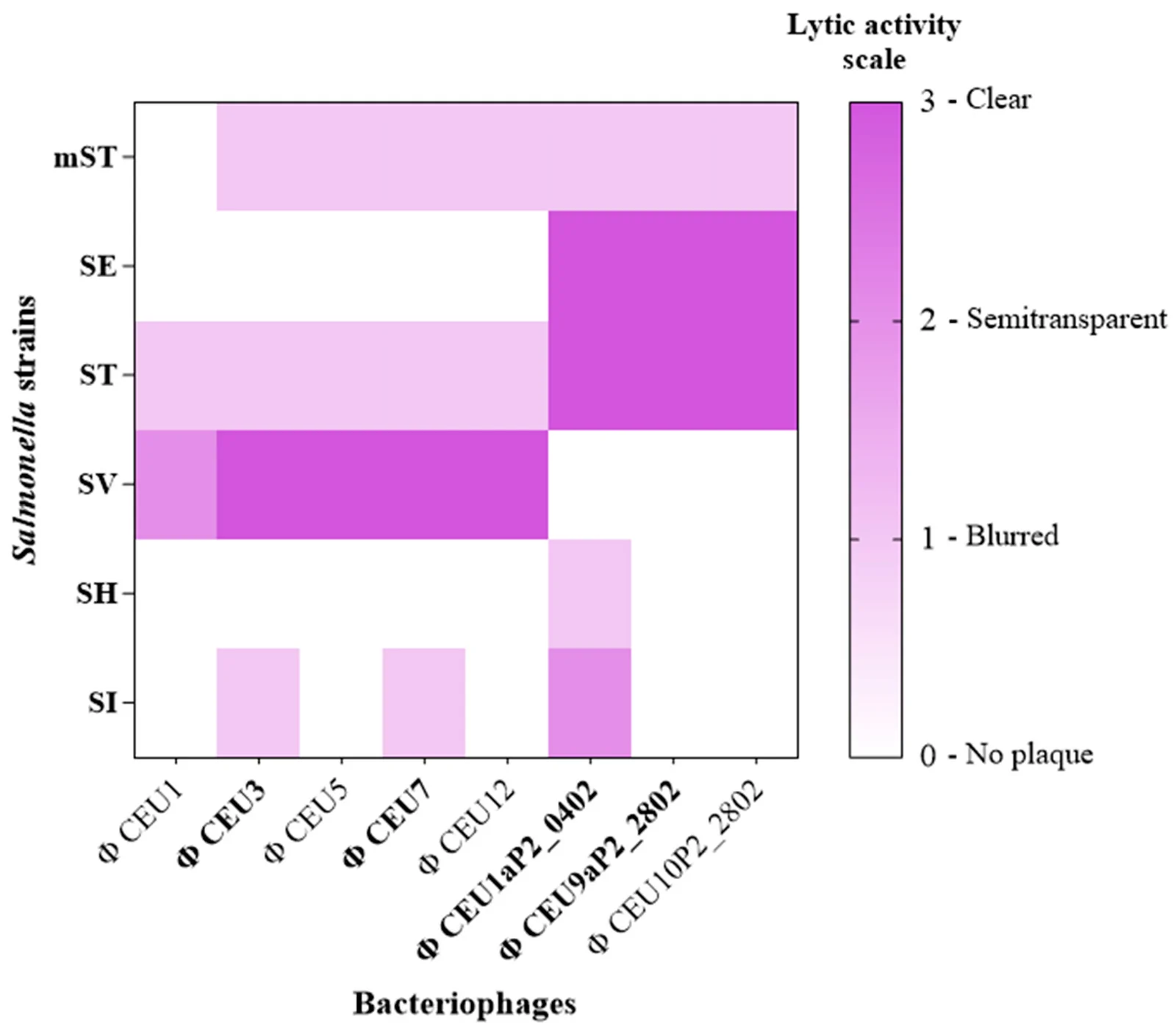
Heatmap showing the lytic capacity of BPs against target *Salmonella* strains. The lytic activity is expressed based on the intensity of lysis observed within the spotted area using a scale ranging from 0 to 3 (0 = no plaque, 1 = blurred, 2 = semitransparent, and 3 = clear). Colour intensity is proportional to lytic capacity. Rows represent *Salmonella* strains: monophasic *Salmonella* Typhimurium (mST), *Salmonella* Enteritidis (SE), *Salmonella* Virchow (SV), *Salmonella* Hadar (SH) and *Salmonella* Infantis (SI), while columns represent the tested BPs.

**Figure 2:**
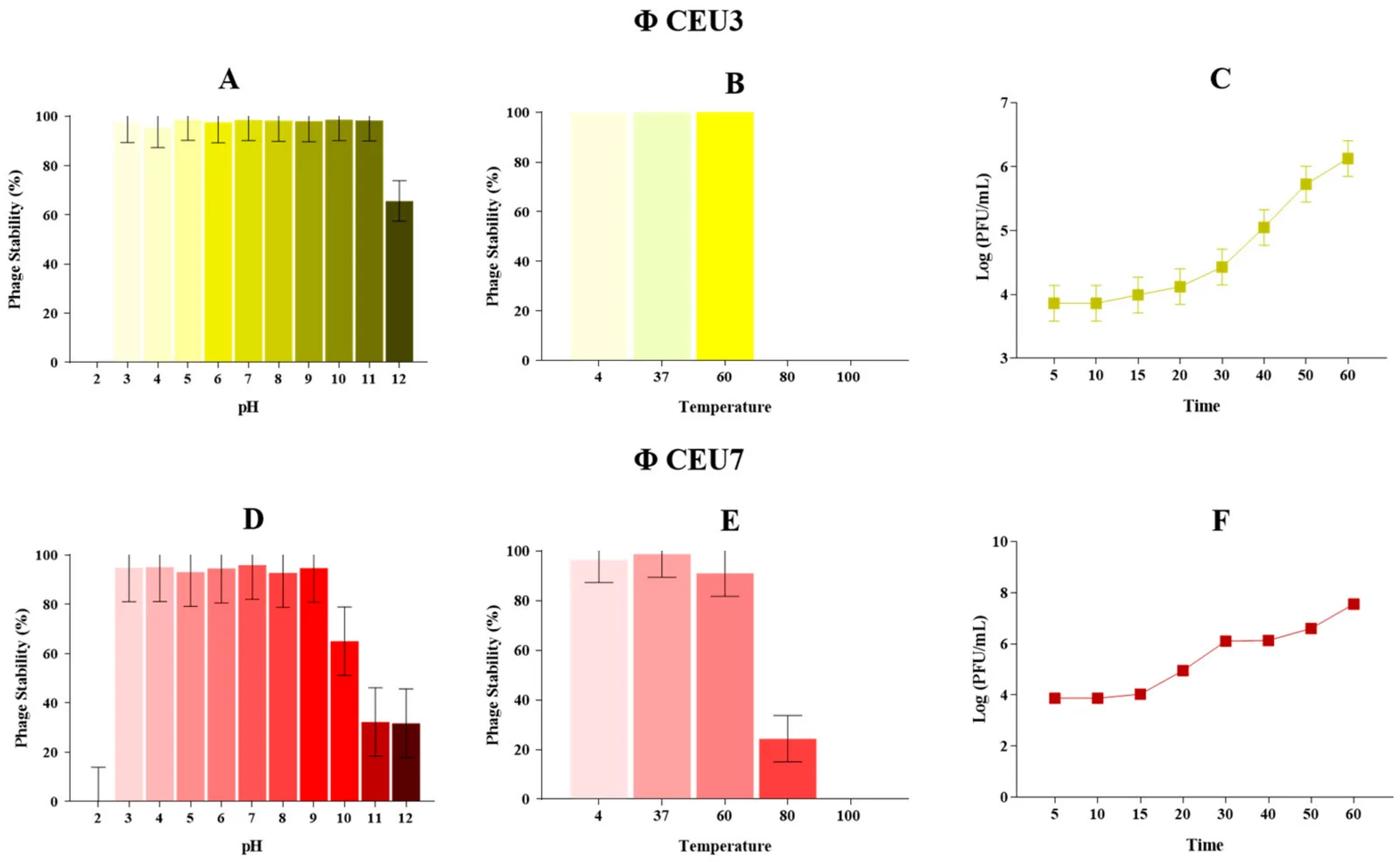
Stability of the BPs under different stress conditions and one-step growth curves. pH stability (**A**), thermal stability (**B**), and one-step growth assay (**C**) for Φ CEU3; pH stability (**D**), thermal stability (**E**), and one-step growth assay (**F**) for Φ CEU7. Data are presented as means ± standard error from three replicates of each experiment. Error bars indicate the standard error of the mean.

**Figure 3:**
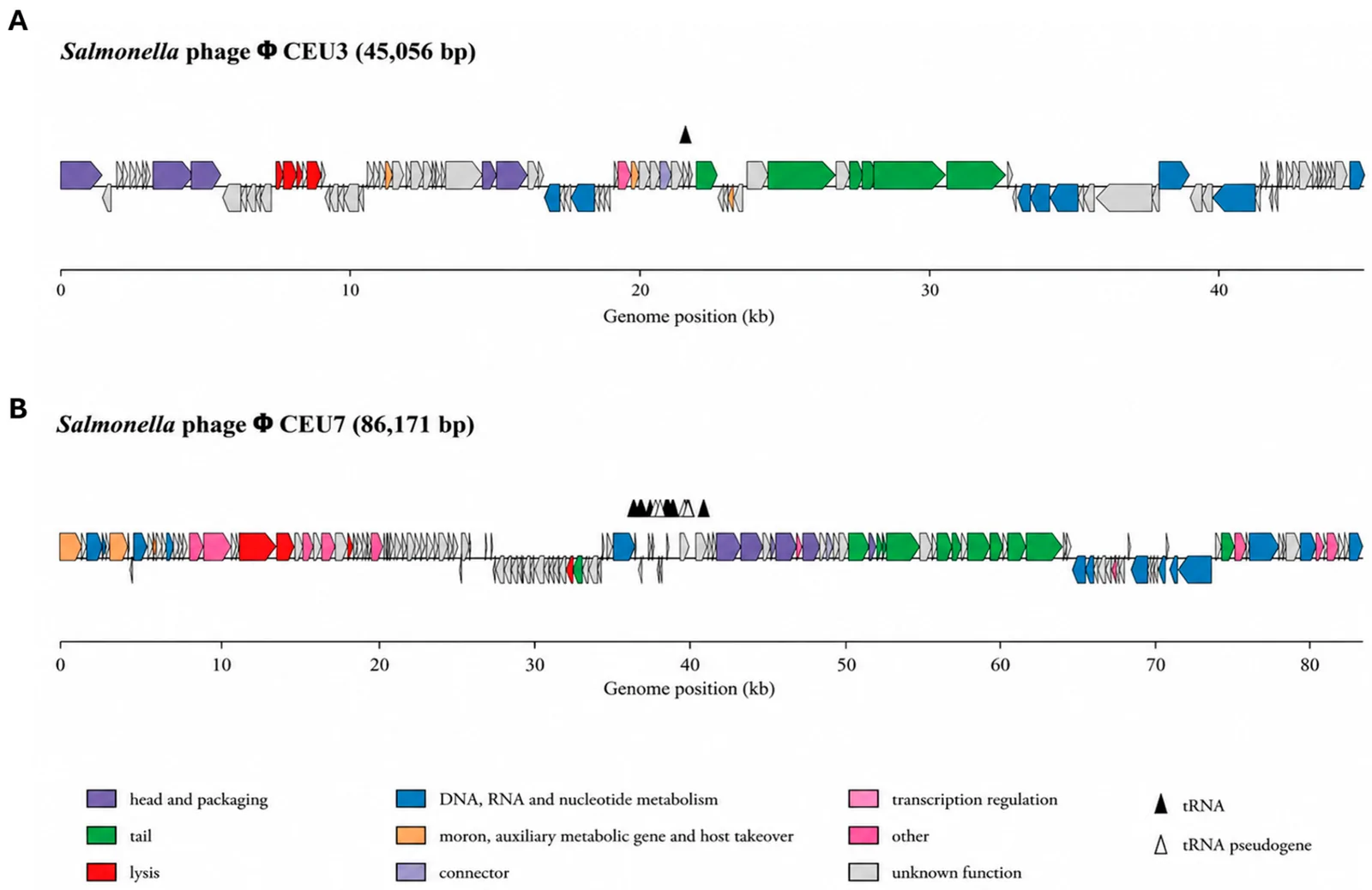
Genome map of *Salmonella* phage Φ CEU3 and Φ CEU7. (**A**) Φ CEU3 and (**B**) Φ CEU7. Predicted CDSs are represented by arrows indicating their transcriptional orientation and are coloured according to the functional categories assigned by Pharokka. tRNAs are indicated by black triangles, whereas predicted tRNA pseudogenes are indicated by open triangles.

**Figure 4:**
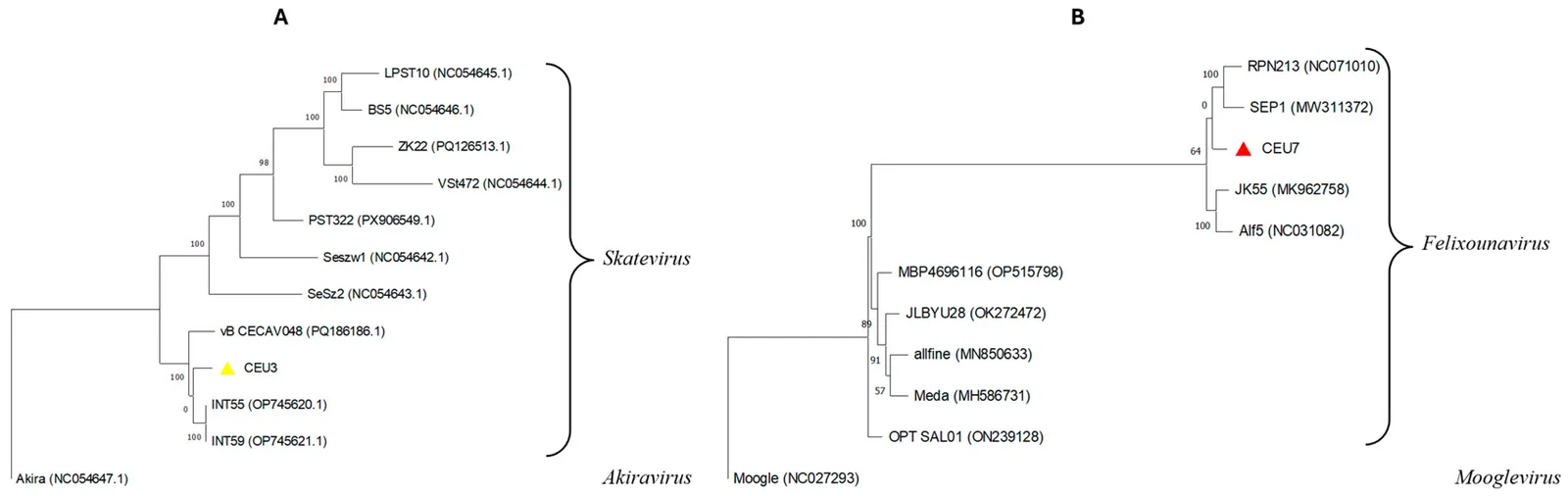
Whole-genome phylogenetic relationships of *Salmonella* phage Φ CEU3 and Φ CEU7. (**A**) Phylogenetic analysis of Φ CEU3, and (**B**) Phylogenetic analysis of Φ CEU7. Branch support values are shown at the nodes. Φ CEU3 and Φ CEU7 are highlighted by yellow and red triangles, respectively. *Salmonella* phage Akira (NC054647.1) and *Citrobacter* phage Moogle (NC027293) were used as outgroups for Φ CEU3 and Φ CEU7, respectively.

**Figure 5:**
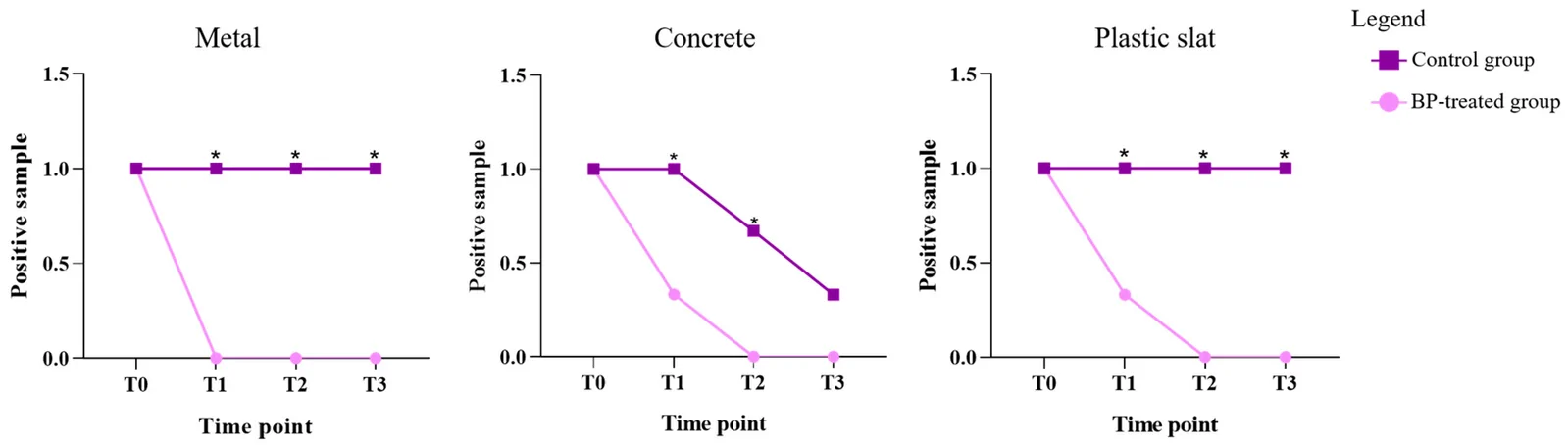
*Salmonella* detection on poultry faeces-contaminated surfaces (metal, concrete and plastic slat) over time under control and BP-treated conditions. T0: 3 h after surface contamination and before the first BP cocktail application; T1: 24 h after the first application of the BP cocktail and before the second application; T2: 24 h after the second application of the BP cocktail; T3: 5 days after the second application of the BP cocktail. Data are expressed as means ± standard error from three replicates. * Significant differences between the groups.

**Figure 6:**
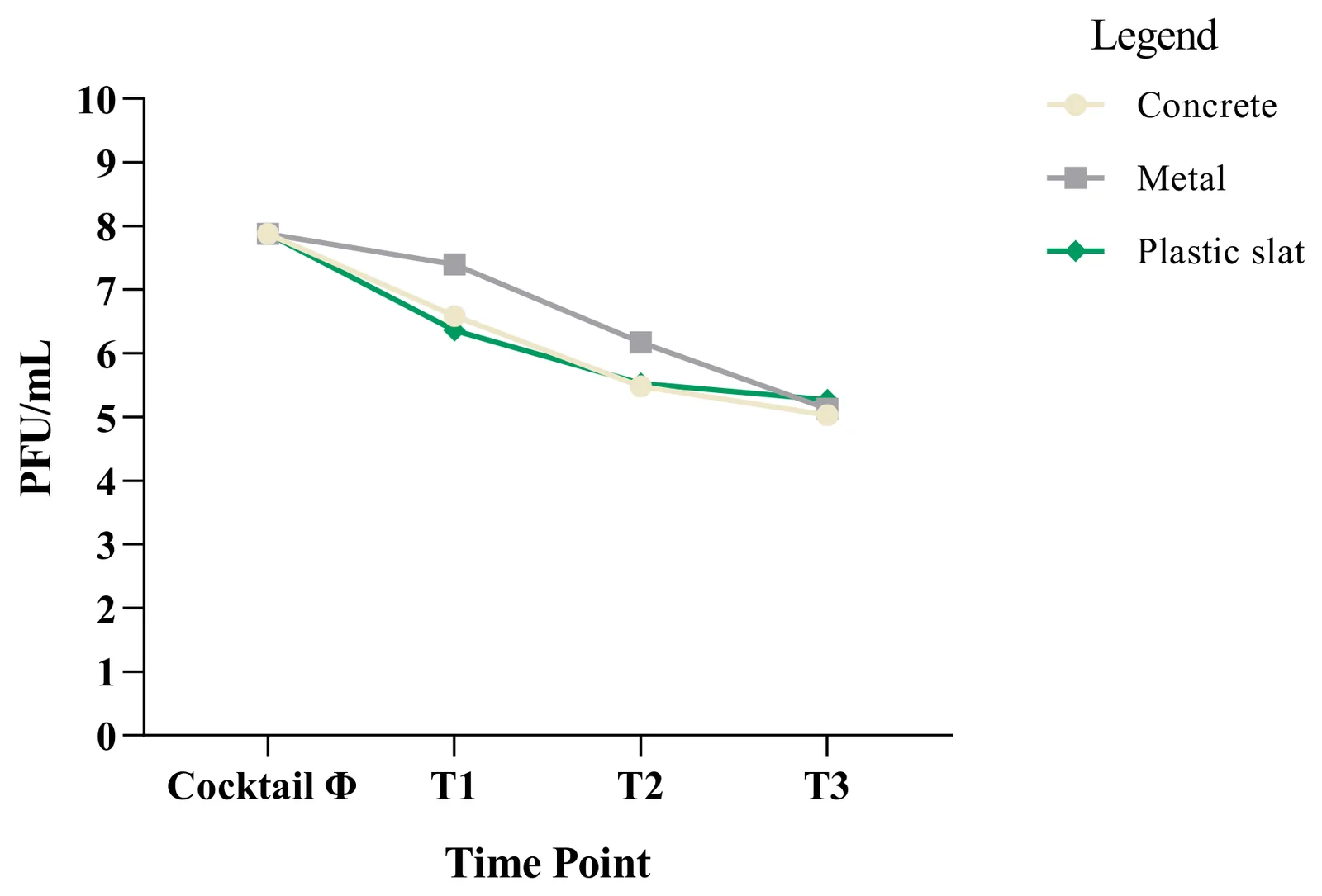
Stability of BP cocktail on surfaces contaminated with poultry faeces, artificially contaminated with monophasic *Salmonella* Typhimurium (mST) over time. Data are expressed as the log_10_-transformed BP cocktail concentration (PFU/mL) on the different surfaces at each sampling time point. Cocktail Φ: BP cocktail diluted with water and applied on surfaces; T1: 24 h after the first application of the BP cocktail and before the second application, T2: 24 h after the second application of the BP cocktail, and T3: 5 days after the second application of the BP cocktail.

**Figure 7:**
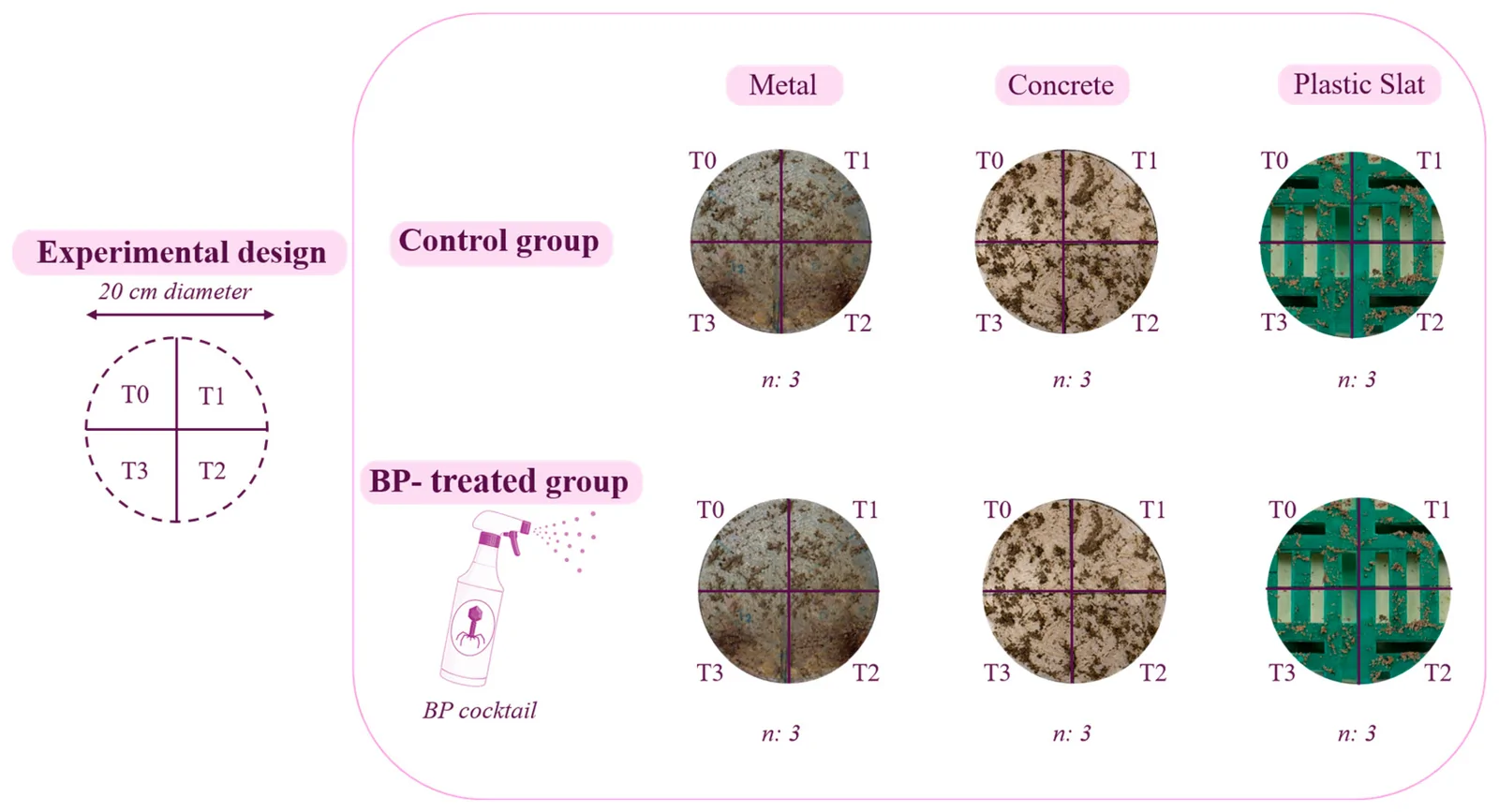
Schematic representation of the experimental design. The schematic representation on the left illustrates the division of the experimental area on each experimental surface: a circular area 20 cm in diameter was delineated and then divided into four equal sampling sections (T0–T3). T0: 3 h after surface contamination and before the first BP cocktail application; T1: 24 h after the first BP cocktail application and before the second application; T2: 24 h after the second BP cocktail application; T3: 5 days after the second BP cocktail application. The upper section of the figure shows the untreated control group, whereas the lower section shows the BP-treated group. Both groups included metal, concrete, and plastic slat surfaces, with the BP cocktail applied only to the BP-treated group. For each surface type and treatment condition, three independent surfaces were used as biological replicates (*n* = 3).

**Table 1:** Antimicrobial resistance profile of multidrug-resistant *Salmonella* strains.

ID *Salmonella* Strain	AMR	MDR	N°
mST (CEU-215S)	SME-AMP-TET	PEN-FOL-TET	3
SV (CEU-598S)	CTZ-TIG-CIP-NAL	CEF-QUI-GLI	3
SI (CEU-582S)	SME-TRI-CIP-NAL-TET	QUI-FOL-TET	3

mST: monophasic *Salmonella* Typhimurium; SV: *S*. Virchow; and SI: *S.* Infantis. AMR: Antimicrobial resistance. CTZ: ceftazidime; SME: sulfamethoxazole; TRI: trimethoprim; TIG: tigecycline; AMP: ampicillin; CIP: ciprofloxacin; NAL: nalidixic acid; TET: tetracycline. MDR: Multidrug resistance. PEN: penicillins; FOL: folate pathway inhibitors; CEF: cephalosporins; QUI: quinolones; and GLI: glycylcyclines. N°: Number of antimicrobial classes to which each *Salmonella* strain is resistant.

**Table 2:** CEU-UCH phage library.

Bacteriophages (Φ)	Propagation Strain of Φ	Origin of Φ
Φ CEU1	CEU-671S	Swine farm sewage water
Φ CEU3	CEU-671S	
Φ CEU5	CEU-582S	
Φ CEU7	CEU-582S	
Φ CEU12	CEU-40S	
Φ CEU1P2_0402	CEU-5S	Urban sewage water [32]
Φ CEU9P2_2802	CEU-92S	
Φ CEU10P2_2802	CEU-97S	

## Data Availability

The datasets used during this study are publicly available in the RiuNET Repository of the Universitat Politècnica de València (UPV) at https://riunet.upv.es/handle/10251/238701 (accessed on 23 September 2026). In addition, the complete genome sequences of Φ CEU3 and Φ CEU7 have been deposited in GenBank under accession numbers **SUB16421258** and **SUB16421315**, respectively.

## Abbreviations

The following abbreviations are used in this manuscript:

AMI	Amikacin
AMP	Ampicillin
AMR	Antimicrobial Resistance
AZI	Azithromycin
BIC	Bayesian Information Criterion
BPs	Bacteriophages
BPW	Buffered Peptone Water
CARD	Comprehensive Antibiotic Resistance Database
CDSs	Coding sequences
C&D	Cleaning and Disinfection
CEF	Cephalosporins
CFU	Colony-Forming Unit
CHL	Chloramphenicol
CIP	Ciprofloxacin
COL	Colistin
CTA	Cefotaxime
CTZ	Ceftazidime
DTRs	Direct Terminal Repeats
EUCAST	European Committee on Antimicrobial Susceptibility Testing
FISABIO	Foundation for the Promotion of Health and Biomedical Research of the Valencian Community
FOL	Folate Pathway Inhibitors
GEN	Gentamicin
GLI	Glycylcyclines
GLM	Generalised Linear Model
LB	Luria–Bertani
MDR	Multidrug Resistance
MER	Meropenem
MIC	Minimum Inhibitory Concentration
MOI	Multiplicity Of Infection
MSRV	Modified Semi-Solid Rappaport Vassiliadis agar plates
mST	monophasic *Salmonella* Typhimurium
NAL	Nalidixic Acid
NCPs	National *Salmonella* Control Programmes
OD	Optical Density
PEN	Penicillins
PFU	Plaque Forming Unit
QACs	Quaternary Ammonium Compounds
QUI	Quinolones
SE	*Salmonella* Enteritidis
SH	*Salmonella* Hadar
SI	*Salmonella* Infantis
SME	Sulfamethoxazole
SRA	Sequence Read Archive
ST	*Salmonella* Typhimurium
SV	*Salmonella* Virchow
TerL	Terminase Large Subunit
TET	Tetracycline
TIG	Tigecycline
TRI	Trimethoprim
UPV	Universitat Politècnica de València
VFDB	Virulence Factor Database
